# Vergence and Accommodation Cues in Stereo-Localization during the Small-In Large-Out (SILO) Effect

**DOI:** 10.3390/vision6040063

**Published:** 2022-10-18

**Authors:** Marc Argilés, Genis Cardona, Sandra Hosa-Vila, Bernat Sunyer-Grau

**Affiliations:** Department of Optics and Optometry, Universitat Politècnica de Catalunya, E08222 Terrassa, Spain

**Keywords:** vision therapy, SILO effect, *AC/A* ratio, stereo-localization, vectograms

## Abstract

A typical procedure in vision therapy is the use of Quoits vectograms to train fusional vergence ranges by improving stereo-localization, which is the ability to correctly locate the target stimulus in space. With this procedure, the Small-In Large-Out (SILO) effect is usually reported in patients with normal binocular vision and accommodation. In this study, the influence of vergence and accommodation cues, as determined with the accommodative-convergence over accommodation (*AC/A*) ratio, to correctly locate the Quoits vectograms in space was investigated. Twenty participants, aged 29.2 ± 2.8 (mean ± standard deviation) years, without amblyopia or strabismus, were recruited. A geometrical formula was obtained to calculate the theoretical distance to the target stimulus for different vergence demands. Theoretical values were compared with measured distances to the perceived stimuli and stereo-localization accuracy was determined. Stereo-localization accuracy was significantly worse at 10∆ Base In vergence demand (*p* < 0.001). A statistically significant positive correlation was found between *AC/A* ratio and stereo-localization accuracy (i.e., worse accuracy) at 10Δ Base Out vergence demand (rho = 0.446, *p* = 0.049). These findings highlight that *AC/A* ratio may be a secondary cue for stereo-localization when using vectograms in which the SILO effect is manifest. These results assist in the understanding of the physiological basis of vision therapy procedures.

## 1. Introduction

Vision therapy has been employed in optometry to effectively improve binocular dysfunctions such as convergence insufficiency [1,2,3,4] and accommodative insufficiency [5]. Target blur, disparity, and proximity may be minutely altered through visual therapy to normalize the accommodative system, the vergence system and their interactions [6,7]. These procedures can be conducted in monocular (closed-loop in vergence), or dual open-loop conditions, in which both vergence and accommodation loops are active. The interaction between these two systems is known as the accommodative-convergence over accommodation (*AC/A*) and convergence-accommodation over convergence (CA/C) ratios [8].

Vision therapy procedures commonly use vectograms or anaglyphs targets for vergence therapy, both during convergence (base out) and divergence (base in) [9,10]. Vectograms consist of two separate transparent charts containing a printed picture, such as a circular target or Quoit, which is shaped like a rope as in the quoit game. When viewed through polarized filters, each image is presented to one eye only. 

Initially, both images are presented superimposed, a situation in which binocular disparity is absent. When the two images are slid apart, disparity appears and either convergence (crossed fixation) or divergence (uncrossed fixation) are induced. Under these conditions, the Small-In, Large-Out (SILO) effect is perceived by the observer: in convergence, the image appears to be smaller and closer to the observer (Small-In), whereas in divergence the image appears to be larger and farther away (Large-Out). 

Localization in the space of the circle presented in the vectograms is a valuable feedback cue in vision therapy in which patients are asked to place a pointer where they perceive the circle [8]. Perception of the SILO effect might depend on size constancy, which is linked to the accommodation and convergence response [10]. Indeed, vergence and accommodative cues are believed to play an important role in the perception of the SILO effect. The perceptual system makes corrections to maintain size constancy in the presence of opposite changes in retinal image size. For instance, when looking at a distance, in divergence, the retinal image size decreases and the perceptual system compensates this reduction by enlarging the image. The opposite occurs when looking at near, in convergence. Some individuals report perceiving a different effect, the Small-Out, Large-In (SOLI) effect, which might be caused by the observer relying solely on changes in retinal image size, ignoring vergence and accommodation cues [9].

The implications of the SILO effect on stereo-localization, and the role of vergence and accommodative cues in this response in patients with normal binocular vision and accommodation have not been investigated in depth. Therefore, the aim of this study was to determine the contribution of vergence and accommodative cues, measured by the *AC/A* ratio, in stereo-localization accuracy during the SILO effect with vectograms. For this purpose, the theoretical distance of the SILO effect was calculated using linear geometry and compared to the distances perceived by participants in different convergence and divergence demands. The relationship of the discrepancies between both sets of values (distance error or stereo-localization accuracy) and the *AC/A* ratio was analyzed to assess the influence of vergence and accommodative cues. 

## 2. Materials and Methods

### 2.1. Study Sample and Baseline Examinations

Participants were recruited during the month of July 2021 from the university student population and personal networks of the authors. All participants were informed of the purpose of the study and the nature of the tests and signed an informed consent prior to the start of the study. The study was approved by an institutional review board (UPC) and conducted according to the tenets of the declaration of Helsinki.

All participants fulfilled the Sheard criteria for exophoria [11], and the Percival criteria for esophoria [12], and had uncorrected visual acuity (VA) of 6/6 or better at both far and near distances. Participants with presbyopia, amblyopia and strabismus were excluded from the study. All participants had good ocular health and did not take any medication that could influence their visual performance.

A complete ophthalmic examination was conducted before the start of the study consisting of ocular health assessment; VA at distance (6 m) and near (0.4 m); dissociated phorias, evaluated with the Von Graefe method (the median of five repeated measures was obtained); fusional vergence ranges at distance (6 m) and near (0.4 m) with the aid of a phoropter; interpupillary distance (IPD), measured with an autorefractometer (OPD-Scan III, Nidek Co., Ltd., Gamagori, Japan); stereoacuity, assessed with the Random Dot test (placed at 40 cm); near point of convergence (NPC) with the aid of a Royal Air Force rule (the median of three repeated measures was obtained); and amplitude of accommodation, obtained by the minus lenses technique.

### 2.2. Assessment of the SILO Effect

The perceived distance to the stimulus (circle of the Quoit vectograms placed at 0.4 m in front of the observer) during the SILO effect was measured under 5 ∆ Base In (BI), 10 ∆ BI, 5 ∆ Base Out (BO) and 10 ∆ BO demands. At a distance of 0.4 m and zero vergence demand, the stimulus subtended a visual angle of 13.54 degrees. A pointer and measuring tape were used to mark and measure the distance to the perceived location of the stimulus (*d_m_* in convergence or BO conditions and *d’_m_* in divergence or BI conditions). In BO conditions participants held the pointer themselves, whereas in BI conditions an assistant held the pointer and moved it closer and further away until the observer noted it was pointing to the perceived location of the stimulus. For each viewing condition, measurements were repeated three times and the average was obtained for further analysis of the perceived distances. All measurements were conducted using a chin and head rest to ensure consistency in head position. A training session was scheduled before the start of the actual measurements to familiarize participants with the test and with the perception of the SILO effect.

**Figure 1:**
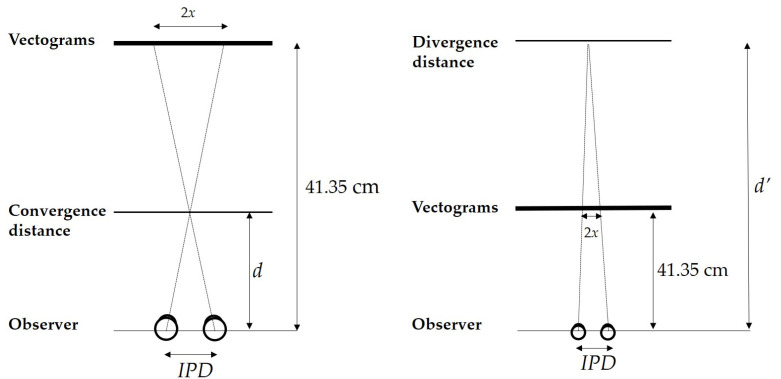
Geometrical calculation of the Small-In, Large-Out (SILO) effect (distance to the perceived stimuli). Similar triangles were defined to determine the theoretical distance in convergence (Small-In) and divergence (Large-Out). *IPD* = Interpupillary distance; *D* = Distance from the center of rotation of the eye to the vectogram; *d* = distance measured in convergence; *d’* = distance measured in divergence; *x* = half the separation distance between the two circular targets (figure not to scale).

Linear geometry was employed to calculate the theoretical distance (*d_s_* in convergence conditions and *d’_s_* in divergence conditions) to the perceived stimulus (Figure 1), as determined by the following equations:

*d_s_ = (41.35 *×* IPD/2)/((IPD/2) + x) d’_s_ = (41.35 *×* IPD/2)/((IPD/2) − x)* (1)

where *IPD* is the interpupillary distance for each participant (in cm), and 2*x* is the separation distance of the vectograms: 2 cm for a 5∆ demand and 4 cm for a 10∆ demand. 

For the calculation, *D* was the distance from the center of rotation of the eye to the vectograms (constant at 40 cm). The center of rotation of the eye is considered to be 1.35 cm posterior to the corneal apex [13,14]. All measurements were recorded in cm. Distances were then referenced to the plane of the vectograms as follows:

*m = 41.35 − d_m_* → *m’ = d’_m_ − 41.35* and → *s = 41.35 − d_s_* → *s’ = d’_s_ − 41.35* (2)

where *m* and *m’* correspond to measured distances in convergence and divergence, respectively, and *s* and *s’* to calculated distances in convergence and divergence, respectively.

Finally, the calculated and measured distances were compared (*s* − *m* for convergence and *s’* − *m’* for divergence) to determine the distance error values, or stereo-localization accuracy (*e_s_*) for each vergence demand. 

### 2.3. Assessment of AC/A Ratio

The *AC/A* ratio was determined with the following equation [7]:

*AC/A = IPD + 0.4 (F_n_ − F_f_)* (3)

where *IPD* is the interpupillary distance for each participant in cm, *F_n_* phoria in near vision (0.4 m) and *F_f_* phoria at distance (6 m), in prism diopters (Δ). Esophoria was expressed in positive values and exophoria in negative values. Therefore, *AC/A* units are Δ/D.

### 2.4. Statistical Analysis

Statistical analysis was performed using SPSS Statistics for Windows, Version 27.0 (IBM Corp, Armonk, NY, USA). Data distribution was explored using Shapiro–Wilk test. Descriptive statistics are summarized with either mean and standard deviation or median and interquartile range. When comparing more than 2 groups of variables, the homogeneity of variance was investigated using Levene’s test of sphericity, and parametric (ANOVA) or non-parametric (Kruskal–Wallis) tests were employed, with the corresponding post-hoc analysis. Similarly, correlation analysis was performed with the Pearson or Spearman correlation coefficients. A *p*-value of 0.05 (α = 0.05, β = 0.95) was considered as the cut-off of statistical significance.

## 3. Results

### 3.1. Study Sample Characteristics

The final sample consisted of 20 participants (*n* = 20) with an age of 29.2 ± 2.8 years (mean ± standard deviation), ranging from 25 to 35 years. Baseline optometric parameters of the study sample values are summarized in Table 1.

### 3.2. Analysis of the SILO Effect

Measured and calculated distances to the stimuli for each vergence demand are shown in Table 2, as well as the values of the corresponding distance errors (*e_s_*). Overall, *e_s_* values were larger, or stereo-localization accuracy worse, in divergence than convergence conditions (*e_s_* 5∆ BO = 1.43 ± 1.41 cm; *e_s_* 10∆ BO = 1.50 ± 1.71 cm; *e_s_* 5∆ BI = 3.65 ± 3.73 cm; *e_s_* 10∆ BI = 13.80 ± 13.87 cm). Statistically significant differences were found between vergence demands (F = 14.015; *p* < 0.001) which a post-hoc analysis with the Bonferroni test revealed to originate between the vergence demand of 10∆ BI and all the other vergence demands (all *p* < 0.001). The vergence demands of 5∆ BO, 10∆ BO and 5∆ BI did not show any statistically significant difference among them (all *p* < 0.05). 

**Figure 2:**
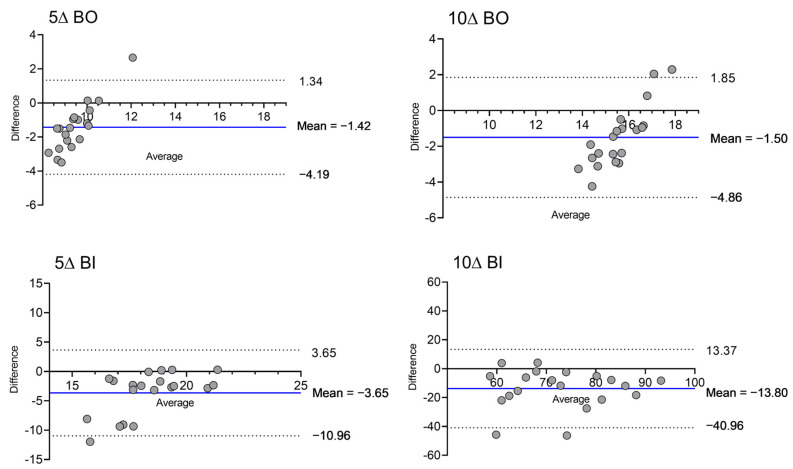
Bland–Altman plot of the differences between theoretical (*s* or *s’*) and measured (*m* or *m’*) distances to the target stimuli for each vergence demand (BO: Base out; BI: Base in). For visualization purposes, axes are not drawn at the same scale. Individual data points are shown as full bullets.

Figure 2 displays the Bland–Altman plots of the pairs *m* and *s* (or *m’* and *s’*) for each vergence demand. 

### 3.3. Correlation Analysis of Stereo-Localization Accuracy

**Figure 3:**
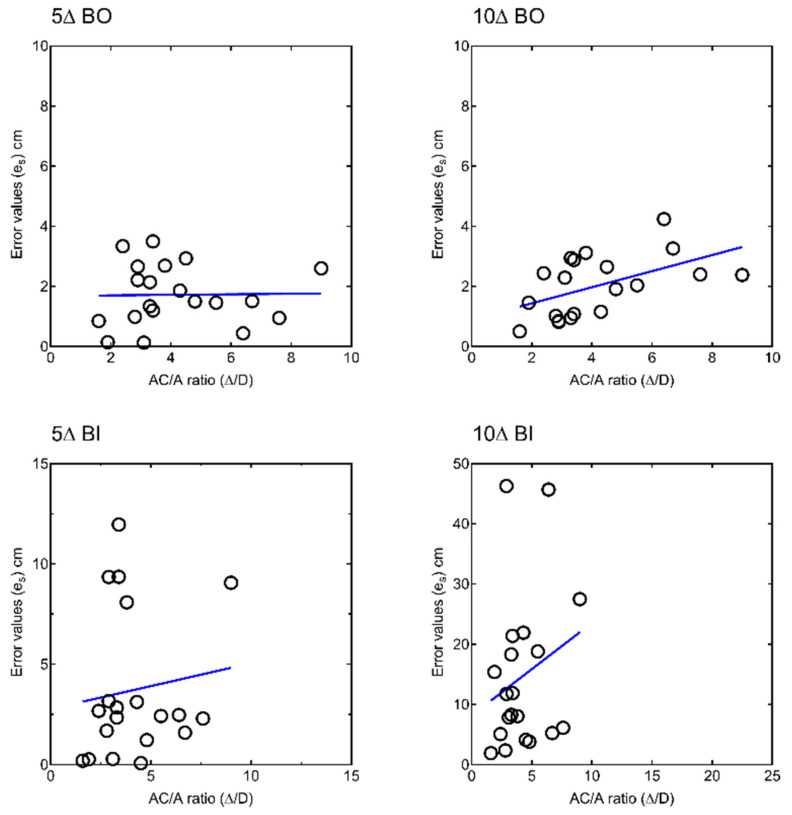
Correlation of accommodative-convergence over accommodation (*AC/A*) ratio and stereo-localization accuracy (*e_s_*) for each vergence demand (BO: Base out; BI: Base in). For visualization purposes, axes are not drawn at the same scale. Individual data points are shown as empty bullets.

A statistically significant positive correlation was found between the *AC/A* ratio and stereo-localization accuracy at 10Δ BO (ρ = 0.446, *p* = 0.049), that is, stereo-localization accuracy was worse for large *AC/A* values. No other statistically significant correlations between *AC/A* ratio and *e_s_* values were found (Figure 3).

Upon exploring other possible associations between baseline vergence and accommodation parameters and stereo-localization accuracy, a statistically significant correlation was found between *e_s_* at 10Δ BO and near phoria values (ρ = −0.590, *p* = 0.006) and between *e_s_* at 5Δ BI and break point BO fusional vergence range (ρ = −0.560, *p* = 0.010). No other statistically significant associations were found between e_s_ and baseline parameters.

## 4. Discussion

The use of vectograms in vision therapy is a common procedure to improve vergence fusional ranges and step vergences [1,2,3,4,7]. In natural conditions (dual closed loop), multiple monocular and binocular cues, such as target blur, disparity, and proximity, elicit accommodative and vergence responses [15]. Vergence and accommodation cues may play an important role in stereo-localization and, as such, may influence the SILO effect. However, the exact mechanisms governing the relationship between vergence and accommodation cues, and stereo-localization accuracy have not been described in the literature, and no mathematical analysis been conducted to explore this effect. The aim of this study was to explore the relationship between the *AC/A* ratio and the accuracy in a stereo-localization task using vectograms in which the perceptual SILO effect was elicited. Stereo-localization accuracy was determined by the difference between the perceived distance to the target stimulus and the distance obtained through geometrical calculation in the absence of accommodation and vergence cues.

Stereo-localization accuracy was found to be worse under divergence than convergence conditions, with a mean difference of 13.80 cm and 3.65 cm between theoretical and perceived localization of the target stimulus for 10 ∆ BI and 5 ∆ BI, respectively, and 1.50 and 1.43 cm for 10 ∆ BO and 5 ∆ BO, respectively. Previous research has documented differences between convergence and divergence responses [16,17,18]. For instance, Hung and co-workers noted different dynamics for convergence and divergence, in terms of velocity, amplitude and latency, consistent with clinical findings in fusional vergence range assessment, suggesting that neural processing delays and controller pathways are different for convergence and divergence [18]. Other authors have observed asymmetries in phasic and tonic vergence responses between convergence and divergence [16]. Indeed, these findings agree with the observed clinical evidence that training fusional vergence ranges using localization cues with vectograms is more efficient under convergence than divergence conditions. Another interesting feature of convergence and divergence asymmetry was the systematic bias between theoretical and perceived distance values displayed in Figure 2, particularly manifest during convergence (BO) but not so in divergence (BI).

Stereo-localization accuracy was also found to decrease with higher values of the *AC/A* ratio, particularly under 10Δ BO vergence demand. Previous studies have described that elevated *AC/A* ratios in subjects with excess of convergence resulted in asymmetric tonic adaptation and destabilization of the vergence system [8]. However, all participants in the present study fulfilled the Percival and Sheard criteria and, albeit higher *AC/A* ratios are expected with lower exophorias at near than far, a stable interconnection between the vergence and accommodation systems should be assumed. Given the absence of a significant correlation between baseline stereoacuity and stereo-localization accuracy, the encountered influence of *AC/A* ratio values on the performance of the stereo-localization task may give support to the role of the perceptual system and the size constancy theory on the perception of the SILO effect.

Thus, the asymmetry between converge and divergence observed in the present research, and the dependence of stereo-localization accuracy on the *AC/A* ratio are relevant in vision therapy, particularly when working with vectograms eliciting the SILO effect. Singh and co-workers found an improvement rate in stimulus and response *AC/A* ratio after 10 sessions of vision therapy [19], in contrast with the results described by Bratauset and Jennings, using stimulus *AC/A* ratio and gradient method, although improvements were observed in fast and slow vergence mechanisms [20]. These discrepancies may be explained with the high variability in stimulus *AC/A* ratio described by some researchers [21,22,23]. Indeed, *AC/A* ratio stimulus assumes that change in accommodation is equal to the visual demand, whilst *AC/A* ratio is computed from the actual response of the accommodation system. In line with these studies, the results observed in the present study evidenced that the highest accuracy in stereo-localization was found in patients with low-normal *AC/A* ratio.

It is worth noting that anaglyphs are also commonly used in vision therapy, as an alternative to vectograms. However, anaglyphs require red and green filters for their correct visualization, which may induce chromatic imbalance, as opposite to the polarized filters employed when working with vectograms [24]. Thus, for the purpose of the present research, vectograms and polarized filters were used.

This study only included participants with normal accommodation and binocular vision, not the typical patient in vision therapy. In order to understand the mechanisms involved in vision therapy procedures, particularly when using vectograms, future studies comparing the stereo-localization accuracy between normal participants and those presenting binocular and accommodative dysfunctions (for instance those showing a SOLI response) are required. It would also be interesting to explore how modifications in *AC/A* ratio, fusional vergence ranges and other parameters induced by vision therapy could, in turn, alter the perception of the SILO effect.

In conclusion, the present research found different stereo-localization accuracy values under convergence and divergence demands, in agreement with previous studies documenting the asymmetries between these systems. These findings support the need to fully explore these parameters before planning vision therapy using vectograms and relying on the SILO effect and may further the understanding of the mechanisms underpinning vision therapy procedures.

## Figures and Tables

**Table 1:** Baseline optometric parameters. Results are shown as either mean (standard deviation) or median (interquartile range). Esophoria is expressed as positive values and exophoria as negative values.

*AC/A* Ratio (Δ/D)	Near Phoria (0.4 m) (Δ)	Distance Phoria (6 m) (Δ)	Stereoacuity (”)	Near Point of Convergence (cm)	Fusional Vergence Ranges (Δ)	Amplitude of Accommodation (D)
4.18 (1.95)	−6.50 (−8.00, 0.25)	0.00 (−1.00, 1.00)	25.00 (18.25, 30.25)	3.00 (3.00, 6.00)	BO = 24.68 (6.63) BI = 16.42 (4.66)	RE = 6.51 (0.99) LE = 6.48 (1.08)

Accommodative-convergence over accommodation (*AC/A*); Prism Diopter (Δ); Diopter (D); Second of Arc (”); Base Out (BO); Base In (BI); Right Eye (RE); Left Eye (LE).

**Table 2:** Measured and calculated distance to the perceived stimuli under each convergence (*m* and *s*) and divergence (*m’* and *s’*) demand and distance error values (*e_s_*) for each participant. Convergence corresponds to Base Out (BO) and divergence to Base In (BI) prisms.

*AC/A* Ratio (Δ/D)	Measured Distance (*m* or *m’*) (cm)				Calculated Distance (*s* or *s’*) (cm)				Distance Error (*s*−*m*) or (*s’*−*m’*) (*e_s_*) (cm)			
	5Δ BO	10Δ BO	5Δ BI	10Δ BI	5Δ BO	10Δ BO	5Δ BI	10Δ BI	5Δ BO	10Δ BO	5Δ BI	10Δ BI
2.80	9.10	15.20	−18.00	−72.80	10.09	16.22	−19.69	−75.18	0.99	1.02	1.69	2.38
4.50	6.80	13.10	−18.30	−70.30	9.73	15.75	−18.38	−66.16	2.93	2.65	0.08	−4.14
7.60	8.90	13.50	−16.50	−62.80	9.85	15.90	−18.80	−68.92	0.95	2.40	2.30	6.12
6.70	8.00	12.20	−16.00	−56.00	9.51	15.46	−17.60	−61.26	1.51	3.26	1.60	5.26
2.90	8.00	17.20	−17.00	−67.00	10.21	16.38	−20.17	−78.76	2.21	−0.82	3.17	11.76
3.80	7.40	13.10	−11.60	−67.10	10.09	16.22	−19.69	−75.18	2.69	3.12	8.09	8.08
9.00	8.00	14.50	−12.70	−64.40	10.60	16.88	−21.76	−91.89	2.60	2.38	9.06	27.49
3.40	9.40	15.80	−12.40	−70.50	10.60	16.88	−21.76	−91.89	1.20	1.08	9.36	21.39
2.90	13.40	16.20	−13.00	−51.00	10.74	17.05	−22.35	−97.29	−2.66	0.85	9.35	46.29
1.60	9.00	15.40	−19.00	−67.00	9.85	15.90	−18.80	−68.92	0.85	0.50	−0.20	1.92
4.80	7.90	13.40	−16.00	−62.90	9.40	15.31	−17.23	−59.07	1.50	1.91	1.23	−3.83
1.90	10.10	14.60	−19.50	−56.50	9.96	16.06	−19.23	−71.91	−0.14	1.46	−0.27	15.41
2.40	7.00	14.10	−18.00	−77.60	10.34	16.54	−20.68	−82.70	3.34	2.44	2.68	5.10
3.10	10.60	19.00	−21.50	−79.20	10.47	16.71	−21.21	−87.05	−0.13	−2.29	−0.29	7.85
4.30	8.10	14.90	−16.10	−50.00	9.96	16.06	−19.23	−71.91	1.86	1.16	3.13	21.91
3.30	8.60	14.10	−19.50	−79.00	10.74	17.05	−22.35	−97.29	2.14	2.95	2.85	18.29
6.40	9.90	12.30	−18.20	−37.00	10.34	16.54	−20.68	−82.70	0.44	4.24	2.48	45.70
5.50	8.50	18.10	−16.80	−53.10	9.96	16.06	−19.23	−71.91	1.46	−2.04	2.43	18.81
3.40	7.10	14.00	−9.80	−80.00	10.60	16.88	−21.76	−91.89	3.50	2.88	11.96	11.89
3.30	9.40	16.10	−20.00	−89.00	10.74	17.05	−22.35	−97.29	1.34	0.95	2.35	8.29

## Data Availability

The dataset that was used and/or analyzed during this study will be available from the corresponding author upon reasonable request.
